# Supplementary material for: Biofilm Spreading by the Adhesin-Dependent Gliding Motility of Flavobacterium johnsoniae. 1. Internal Structure of the Biofilm
Source: Int J Mol Sci. 2021 Feb 14;22(4):1894. doi: 10.3390/ijms22041894 (PMC7918930; doi:10.3390/ijms22041894)
Supplement: Supplementary file 1 [file ijms-22-01894-s001.zip › ijms-1058259-supplementary.pptx]

## Slide 1
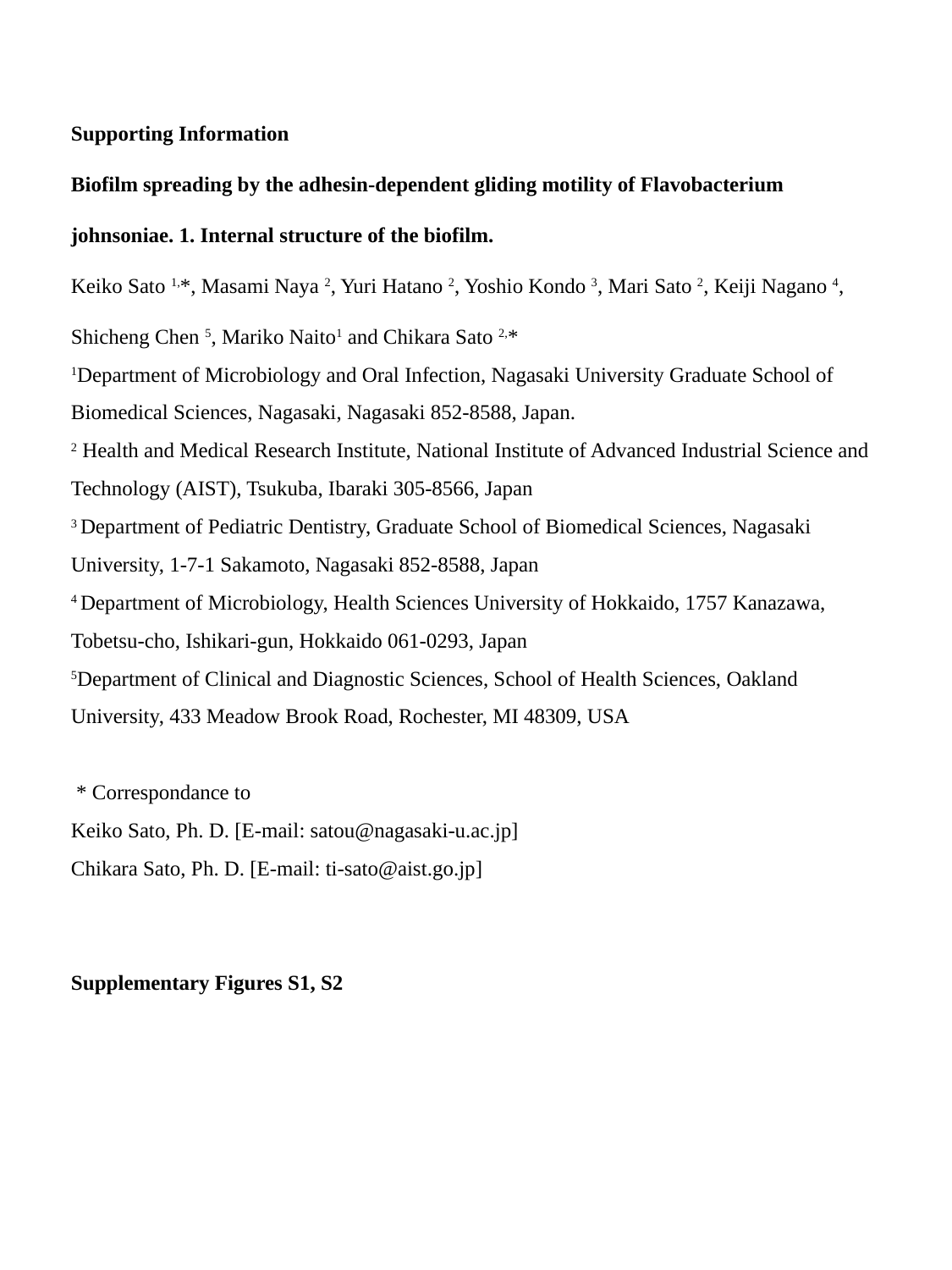

Supporting Information
Biofilm spreading by the adhesin-dependent gliding motility of Flavobacterium johnsoniae. 1. Internal structure of the biofilm.
Keiko Sato 1,*, Masami Naya 2, Yuri Hatano 2, Yoshio Kondo 3, Mari Sato 2, Keiji Nagano 4, Shicheng Chen 5, Mariko Naito1 and Chikara Sato 2,*
1Department of Microbiology and Oral Infection, Nagasaki University Graduate School of Biomedical Sciences, Nagasaki, Nagasaki 852-8588, Japan.
2 Health and Medical Research Institute, National Institute of Advanced Industrial Science and Technology (AIST), Tsukuba, Ibaraki 305-8566, Japan
3 Department of Pediatric Dentistry, Graduate School of Biomedical Sciences, Nagasaki University, 1-7-1 Sakamoto, Nagasaki 852-8588, Japan
4 Department of Microbiology, Health Sciences University of Hokkaido, 1757 Kanazawa, Tobetsu-cho, Ishikari-gun, Hokkaido 061-0293, Japan
5Department of Clinical and Diagnostic Sciences, School of Health Sciences, Oakland University, 433 Meadow Brook Road, Rochester, MI 48309, USA
 * Correspondance to
Keiko Sato, Ph. D. [E-mail: satou@nagasaki-u.ac.jp]
Chikara Sato, Ph. D. [E-mail: ti-sato@aist.go.jp]
Supplementary Figures S1, S2

## Slide 2
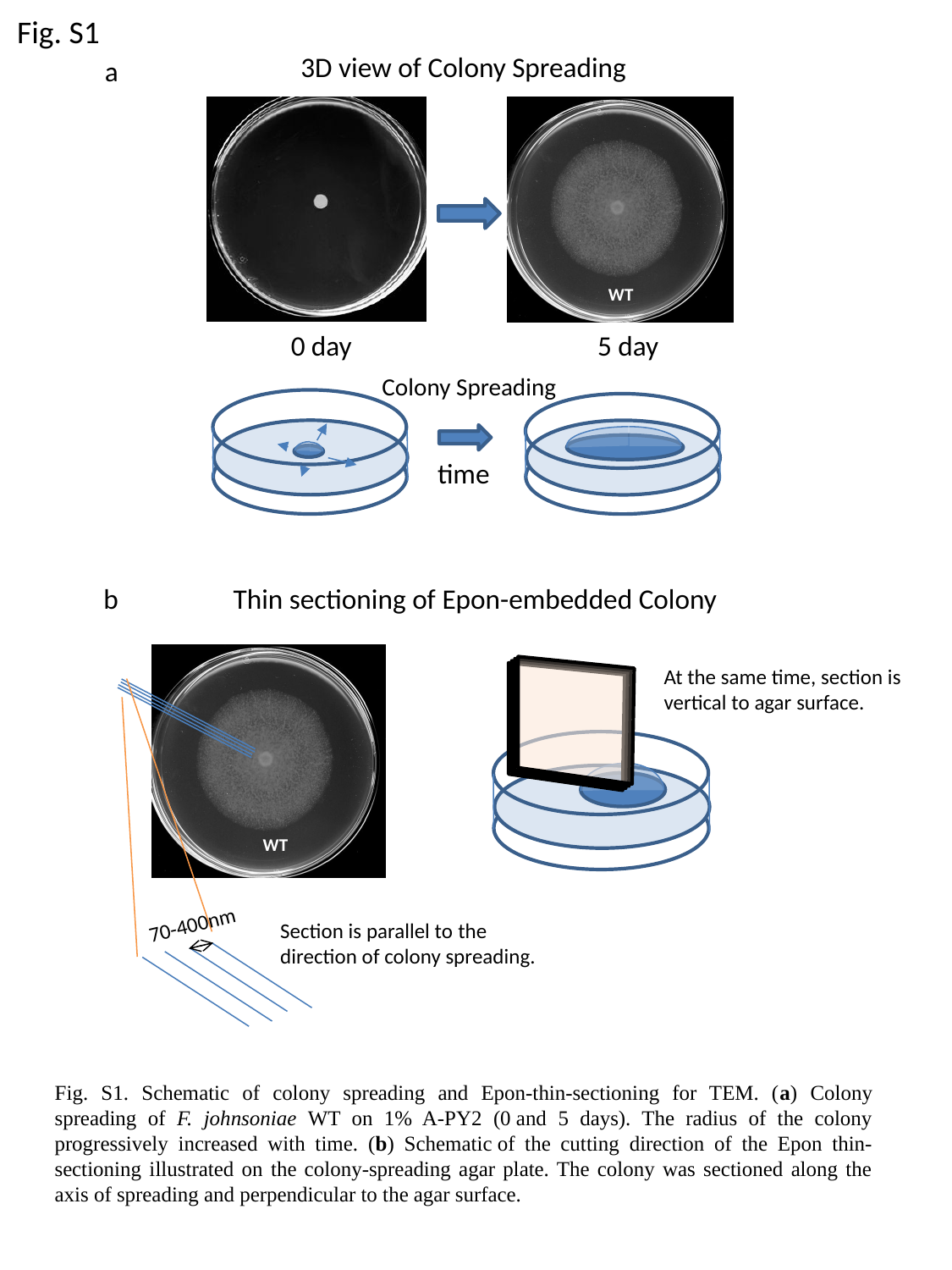

Fig. S1
3D view of Colony Spreading
a
WT
0 day
5 day
Colony Spreading
time
b
Thin sectioning of Epon-embedded Colony
70-400nm
At the same time, section is vertical to agar surface.
WT
Section is parallel to the direction of colony spreading.
Fig. S1. Schematic of colony spreading and Epon-thin-sectioning for TEM. (a) Colony spreading of F. johnsoniae WT on 1% A-PY2 (0 and 5 days). The radius of the colony progressively increased with time. (b) Schematic of the cutting direction of the Epon thin-sectioning illustrated on the colony-spreading agar plate. The colony was sectioned along the axis of spreading and perpendicular to the agar surface.

## Slide 3
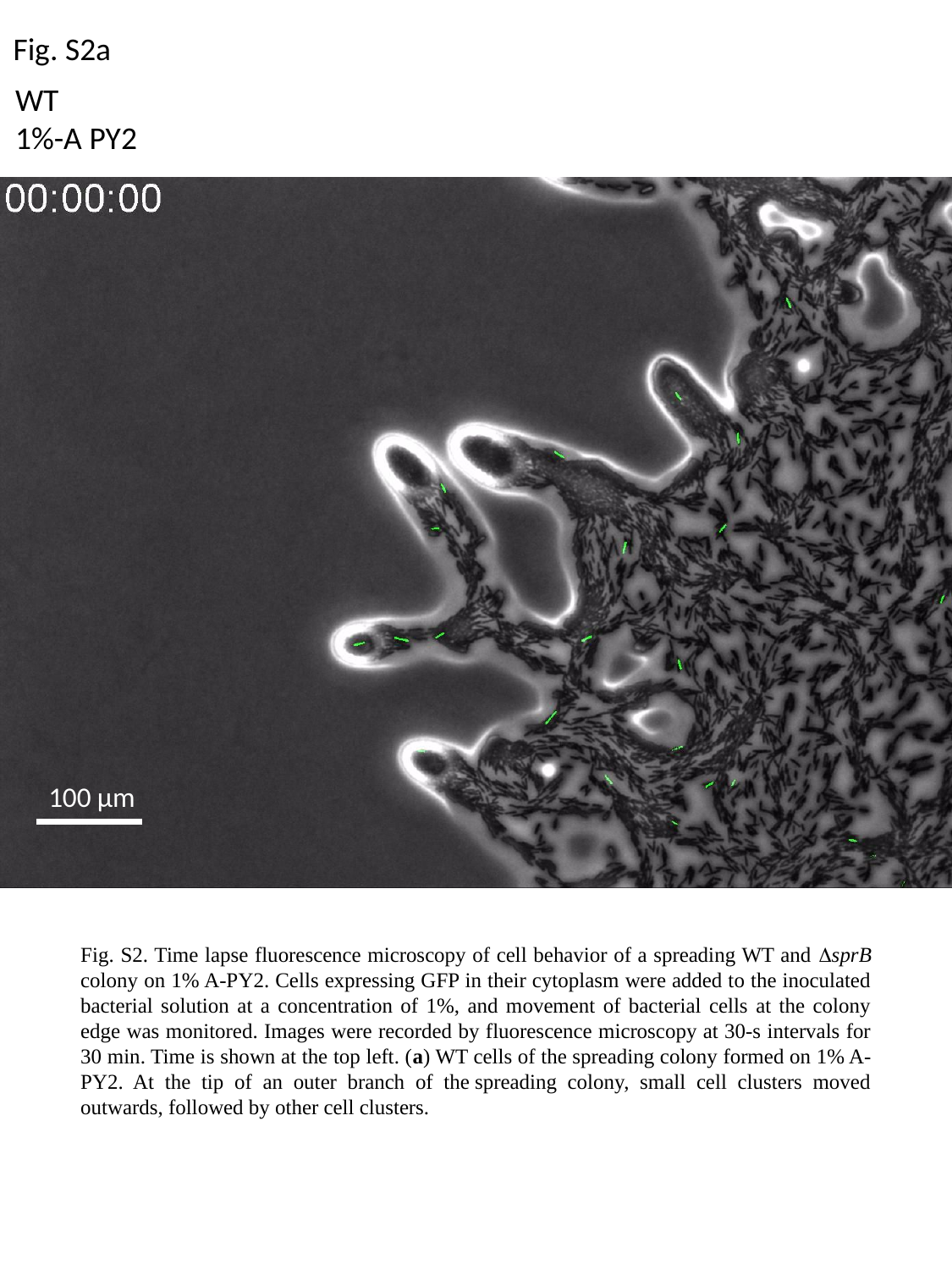

Fig. S2a
WT
1%-A PY2
100 µm
Fig. S2. Time lapse fluorescence microscopy of cell behavior of a spreading WT and DsprB colony on 1% A-PY2. Cells expressing GFP in their cytoplasm were added to the inoculated bacterial solution at a concentration of 1%, and movement of bacterial cells at the colony edge was monitored. Images were recorded by fluorescence microscopy at 30-s intervals for 30 min. Time is shown at the top left. (a) WT cells of the spreading colony formed on 1% A-PY2. At the tip of an outer branch of the spreading colony, small cell clusters moved outwards, followed by other cell clusters.

## Slide 4
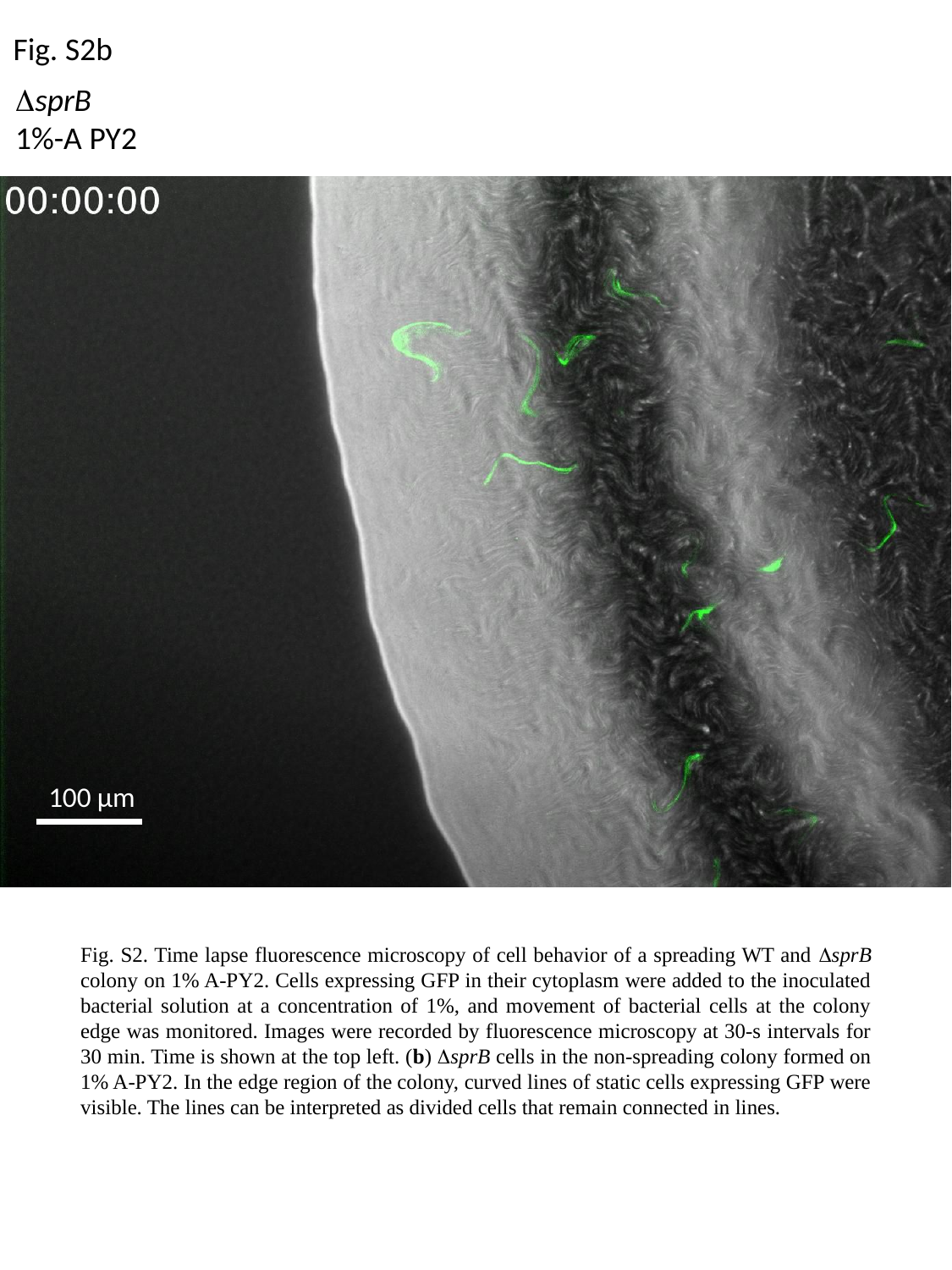

Fig. S2b
DsprB
1%-A PY2
100 µm
Fig. S2. Time lapse fluorescence microscopy of cell behavior of a spreading WT and DsprB colony on 1% A-PY2. Cells expressing GFP in their cytoplasm were added to the inoculated bacterial solution at a concentration of 1%, and movement of bacterial cells at the colony edge was monitored. Images were recorded by fluorescence microscopy at 30-s intervals for 30 min. Time is shown at the top left. (b) DsprB cells in the non-spreading colony formed on 1% A-PY2. In the edge region of the colony, curved lines of static cells expressing GFP were visible. The lines can be interpreted as divided cells that remain connected in lines.
